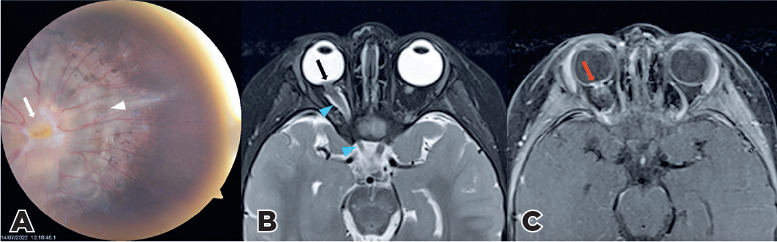# Morning glory disc anomaly and irregular optic nerve thickness: an overlooked association

**DOI:** 10.5935/0004-2749.2024-0330

**Published:** 2025-04-07

**Authors:** Sérgio Ferreira Alves Júnior, Luiz Fernando Teixeira, Paulo Gois Manso, Diogo Goulart Corrêa, Soraya Silveira Monteiro, José Roberto Falco Fonseca

**Affiliations:** 1 Departamento de Diagnóstico por Imagem, Escola Paulista de Medicina, Universidade Federal de São Paulo, São Paulo, SP, Brazil; 2 Departamento de Oftalmologia, Escola Paulista de Medicina, Universidade Federal de São Paulo, São Paulo, SP, Brazil; 3 Departamento de Radiologia, Universidade Federal Fluminense, Rio de Janeiro, RJ, Brazil

A 2-year-old patient had right ocular esotropia for 1.5 years. Right fundoscopy revealed a large disc, central glial tuft (*arrow*), and a halo of pigmentary changes in the peripapillary area surrounding the nerve (arrowhead) consistent with methylglycinediacetic acid (MGDA) (Figure 1A). Funnel-shaped morphology of the optic disc (*black arrow*), adjacent retinal surface elevation and enhancement (*red arrow*), and irregular thickening of the nerve (*blue arrowheads*) were detected (Figure 1B, Figure 1C). Irregular thickness of optic nerve occurs in 89% MGDA, the nature being uncertain^(1^-^3)^.

**Figure:**